# Stratified signaling network remodeling of kinase–transcription factors’ interactions in Parkinson’s disease

**DOI:** 10.1093/bioadv/vbag059

**Published:** 2026-02-17

**Authors:** Xiaoyan Zhou, Luca Parisi, Sicen Liu, Ziqi Cheng, Hanwen Liang, Mansour Youseffi, Farideh Javid, Renfei Ma

**Affiliations:** Faculty of Biology, Shenzhen MSU-BIT University, Shenzhen, Guangdong 518115, China; Department of Computer Science, Tutorantis, Edinburgh, EH2 4AN, United Kingdom; SMBU-MSU-BIT Joint Laboratory on Bioinformatics and Engineering Biology, Shenzhen MSU-BIT University, Shenzhen, Guangdong 518115, China; Faculty of Biology, Shenzhen MSU-BIT University, Shenzhen, Guangdong 518115, China; Faculty of Biology, Shenzhen MSU-BIT University, Shenzhen, Guangdong 518115, China; Faculty of Management, Sciences and Engineering/School of Computing and Engineering, University of Bradford, Bradford, West Yorkshire BD7 1DP, United Kingdom; Department of Pharmacy, University of Huddersfield, Queensgate, HD1 3DH, United Kingdom; Faculty of Biology, Shenzhen MSU-BIT University, Shenzhen, Guangdong 518115, China

## Abstract

**Motivation:**

Understanding how signaling networks differ across molecular subgroups of Parkinson’s disease (PD) is essential for gaining further mechanistic insights and advancing therapeutic development for the disease. This study introduces an integrative, stratified computational framework to characterize subgroup-specific changes in kinase–transcription factors’ (TFs) interactions using transcriptomic profiles.

**Results:**

Differential expression analysis was leveraged to identify kinases with altered expression across various PD subgroups, while transcription factor activity inferred by multi-sample Virtual Inference of Protein-activity by Enriched Regulon revealed dysregulated transcription relative to controls. Phosphorylation data from SIGNOR 4.0 enabled the construction of kinase–TF subnetworks, which were analysed via pathway enrichment to reveal affected biological pathways. Comparative analyses and modeling revealed both shared and distinct signaling features among PD stratified subgroups. A recurring pattern across multiple groups involved STAT family-specific activation downstream of receptor and non-receptor tyrosine kinases, consistently with a conserved inflammatory and pro-survival signaling axis. In contrast, PD_LRRK2 showed selective involvement of immune-metabolic pathways, including AMPK to HNF4A and PAK5 to NF-κB, while PD_GBA and prodromal cohorts were characterized by stress and apoptosis-related mechanisms involving MAPK10 (JNK3), TP53, and hormone receptor pathways (AR and ESR1). Overall, this novel stratified computational framework integrates gene expression, infers subtle TF activity, identifies differentially expressed kinases, and leverages mechanistic interaction data to unveil signaling heterogeneity in PD. Identifying regulators and subgroup-specific network features provides opportunities to inform, influence, and enable the unveiling of novel biomarkers and develop more effective and proactive precision therapeutics.

**Availability and Implementation:**

Source code is available at https://github.com/xyzhou218/Kin_TF_net.

## Introduction

Parkinson’s disease (PD) is a heterogeneous neurodegenerative disorder characterized by progressive loss of dopaminergic neurons, α-synuclein aggregation, mitochondrial dysfunction, synaptic impairment, oxidative stress, and widespread neuroinflammation (Caligiore *et al*. 2016). Although genetic studies have revealed various genes and pathways associated with disease risk (Billingsley *et al*. 2018, Wu *et al*. 2025), these account for only a part of the observed phenotypic diversity. These heterogeneous processes interact via distinct signaling networks, creating unique regulatory patterns across stratified patient subgroups. Therefore, a deeper and more comprehensive understanding of this molecular heterogeneity is crucial for advancing our understanding of the mechanisms and developing mechanism-based therapeutic strategies.

Kinases are key regulators of cellular signaling in many diseases, including PD (Ma *et al*. 2023a). They transduce extracellular stimuli into coordinated transcriptional responses by modulating downstream TFs (Weidemüller *et al*. 2021). This modulation typically involves post-translational modifications that either activate or inactivate TFs, with phosphorylation being the predominant type (Ma *et al*. 2023a, b). Although some kinases have been demonstrated to be related to PD (Wu *et al*. 2025), a broader kinase–TF regulatory architecture is understudied. The regulatory network governing the heterogeneity of PD subgroups still needs further investigation. Dysregulation of different regulatory pathways represents a distinct PD molecular mechanism. For instance, the brain-derived neurotrophic factor (BDNF)/tropomyosin receptor kinase B (TrkB)/cyclic adenosine monophosphate response element-binding protein (CREB) pathway mitigates neurodegeneration and PD-related depression by maintaining neuronal survival and synaptic plasticity (Zhao *et al*. 2021). The phosphoinositide 3-kinase (PI3K)/protein kinase B (AKT) pathway enhances dopaminergic neuronal survival by inhibiting apoptosis and oxidative stress mediated through downstream targets such as the mechanistic target of rapamycin (mTOR) (Long *et al*. 2021). Additionally, both the α-synuclein/Toll-like receptors (TLRs)/nuclear factor kappa-light-chain-enhancer of activated B cells (NF-κB)/NOD-, LRR- and pyrin domain-containing protein 3 (NLRP3) inflammasome axis (Li *et al*. 2021) and the Janus kinase (JAK)/signal transducer and activator of transcription (STAT) pathway (Jain *et al*. 2021) drive chronic neuroinflammation. Meanwhile, the AMP-activated protein kinase (AMPK)/peroxisome proliferator-activated receptor gamma coactivator 1-alpha (PGC-1α) axis maintains energy homeostasis under metabolic stress and promotes mitochondrial biogenesis and antioxidant defense mechanisms (Athari *et al*. 2024). These signaling networks demonstrated that kinases regulate TF-mediated responses heterogeneously, resulting in the pathological progression of PD.

Targeting vital kinases (Raza *et al*. 2025) and transcription factors (TFs), such as the protein kinase C (PKC) family (Odumpatta and Arumugam 2021) and transcription factor EB (TFEB) (Rai *et al*. 2021), has been considered as a potential treatment for PD. Notably, there are many subgroups of PD, as presented in Parkinson’s Progression Markers Initiative (PPMI). There are extensive studies on PD diagnosis and sub-typing (Zhou *et al*. 2025, Parisi *et al*. 2022). The literature on subgroup-specific regulatory networks from a phosphorylation perspective is limited. A comprehensive analysis of subgroup-specific kinase–TF regulatory networks would better understand subgroup heterogeneity and support more precise therapeutic strategies, including the development of kinase- and TF-targeted interventions (Xu *et al*. 2024). However, it is still unclear how kinase-mediated TF regulation varies specifically across stratified PD subgroups at the network level, leaving a crucial gap in understanding the underlying molecular basis of PD heterogeneity. In our previous study (Zhou *et al*. 2025), we developed a comprehensive framework that integrates multi-omics data, including transcriptomics and proteomics, for the diagnosis and subtyping of PD. Using this framework, we identified proteomic features with strong diagnostic potential, many of which were linked to signaling and receptor-mediated pathways. Functional enrichment analysis highlighted significant involvement of the PI3K/AKT signaling pathway, which has been consistently implicated in PD pathogenesis through its roles in oxidative stress regulation, mTOR signaling, and Tau phosphorylation (Zhou *et al*. 2025). These findings underscore the central importance of kinase-mediated signaling in neuronal survival and degeneration, suggesting that dysregulation of signaling proteins may contribute to both molecular and clinical heterogeneity in PD.

As a follow-up to our previous study (Zhou *et al*. 2025), this study aimed to systematically reconstruct stratified subgroup-specific kinase–TF regulatory networks to investigate how upstream kinase perturbations influence downstream transcriptional programs and drive distinct pathogenic mechanisms across PD subgroups. In this study, RNA-seq data obtained from the PPMI were leveraged to analyse and identify signaling regulators that contribute to molecular heterogeneity in PD. Differential expression analysis was first performed to identify kinases with altered transcript levels across PD subgroups compared with healthy controls. Meanwhile, a multiple sample Virtual Inference of Protein-activity by Enriched Regulon analysis (msVIPER) (Alvarez *et al*. 2016) was leveraged to infer subgroup-specific TF activity alterations. Thereafter, these kinases and TFs were leveraged concurrently with curated phosphorylation relationships from the latest version of the SIGnaling Network Open Resource (SIGNOR 4.0) (Lo Surdo *et al*. 2026) to derive candidate kinase–TF regulatory interactions supported by existing phosphorylation evidence. Functional enrichment analyses using Gene Ontology (GO), Kyoto Encyclopedia of Genes and Genomes (KEGG), and Reactome resources were then used to characterize the biological pathways associated with the inferred regulatory relationships. This study highlights molecular mechanisms underlying PD heterogeneity by harnessing the patterns of the resulting stratified subgroup-specific signaling networks and enriched pathways in the context of PD biology and pathophysiology. This research helps to unveil candidate signaling regulators for informing mechanistic and therapeutic interventions.

## Materials

### Dataset

The RNA-seq raw reads used in this study were obtained with approval from the PPMI database (https://www.ppmi-info.org/). RNA-seq counts were used to identify differentially expressed kinase-encoding genes, and normalized expression values in transcripts per million (TPM) were employed for downstream TF activity inference. The PPMI database consists of four cohorts, including PD participants, healthy controls (HC), Scans Without Evidence of Dopaminergic Deficit (SWEDD), and Prodromal. Among them, the SWEDD cohort includes individuals with clinical features suggestive of PD but without evidence of nigrostriatal dopaminergic deficits on DAT-SPECT or PET imaging. Each cohort consists of several subgroups, mainly defined by genes carrying PD-related mutations and clinical phenotypes. Sporadic PD refers to PD cases without a known monogenic cause or a strong family history (Chai and Lim 2013). LRRK2 mutation carriers are individuals with pathogenic LRRK2 variants, which are the most common genetic causes of PD (Tolosa *et al*. 2020). GBA mutation carriers are individuals with pathogenic variants in GBA that cause lysosomal dysfunction and impaired α-synuclein clearance, leading to reduced glucocerebrosidase activity (Smith and Schapira 2022). In contrast, REM sleep behavior disorder (RBD) is considered a prodromal clinical subgroup, with studies indicating that most patients eventually convert to PD, dementia (Anang *et al*. 2014), or multiple system atrophy (Palma *et al*. 2015). Hyposmia participants are defined based on impaired olfactory function, a prevalent early non-motor symptom of PD. For some participants, multiple visits were recorded. Supplementary Table S1 provides a complete summary of all included participants, listing their participant IDs, cohort, subgroup, and total number of recorded visits. As a result, each visit was considered one distinct visiting event in this study. To ensure statistical significance, this study focused on subgroups with sufficient event sizes, excluding those with fewer than 50 recorded visiting events. Subgroups included for downstream analysis, and the number of events for corresponding subgroups, are summarized in Supplementary Table S2.

## Methods

### Schematic of the proposed method

**Figure 1:**
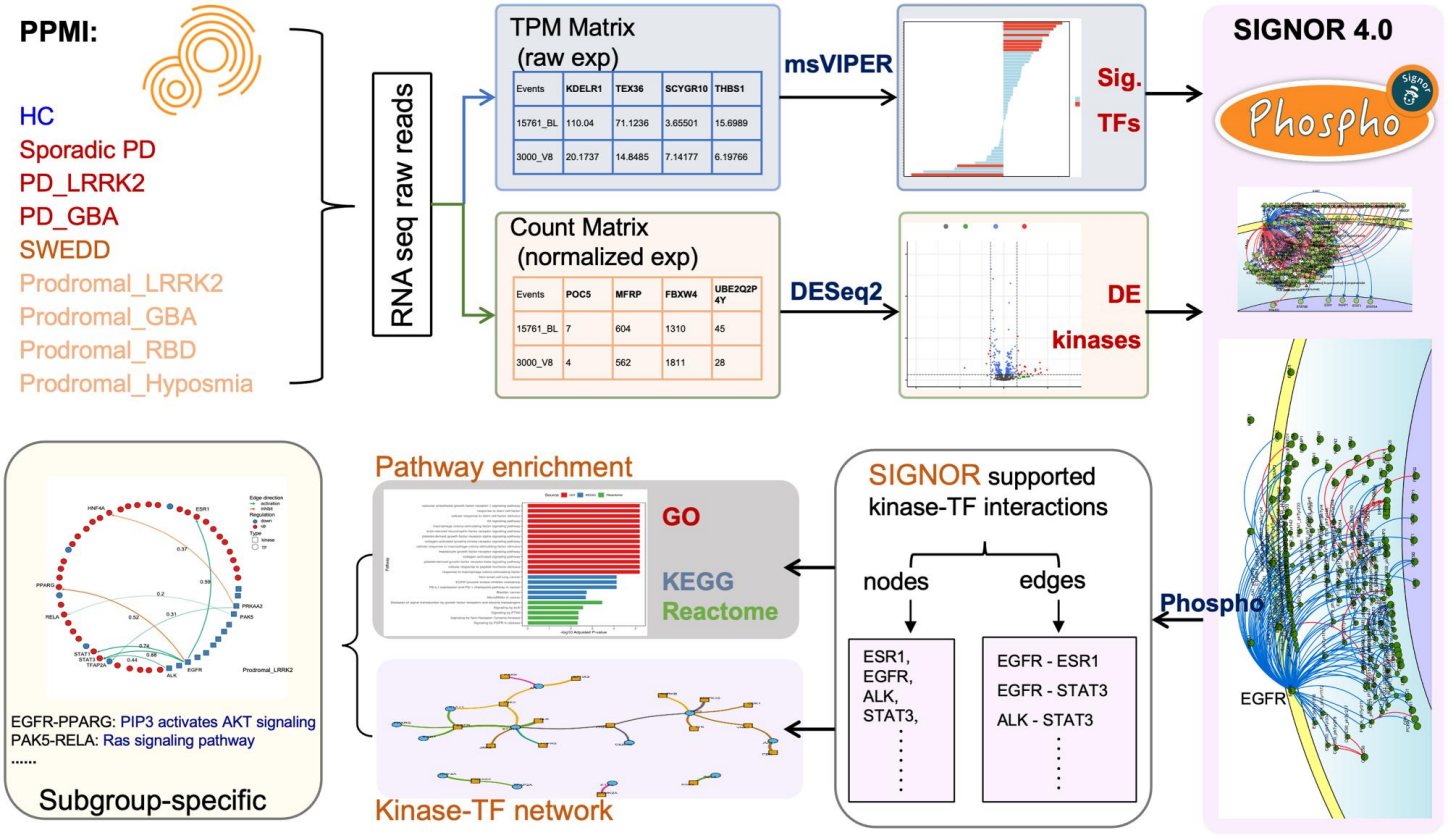
Workflow schematic for analysing kinase–TF interactions across PD subgroups. Significant TFs were inferred using msVIPER, and differentially expressed kinases were identified by differential expression analysis. These TFs and kinases were integrated with SIGNOR 4.0 to retain potential kinase–TF phosphorylation relationships. Subsequently, pathway enrichment was performed, and enriched pathways were combined with kinase–TF links to construct subgroup-specific signaling networks that characterize regulatory alterations in each PD subgroup.

A schematic overview of the analytical workflow is shown in Fig. 1. RNAseq data were represented in two formats, including raw count matrix (integer read counts) and TPM matrix (normalized expression), in PPMI. The count matrix was used for differential expression analysis, while the TPM matrix was employed for downstream regulatory activity inference.

To infer the relative activity levels of TF, this study used the msVIPER algorithm. This method can obtain the relative activity of regulatory genes through the enrichment of their most closely regulated targets on a given gene expression signature (Alvarez *et al*. 2016). Notably, it infers regulator activity from the coordinated expression of downstream transcriptional targets, enabling the detection of regulatory effects that may not be captured by regulator expression levels alone (Alvarez *et al*. 2014). This algorithm generates activity scores by evaluating the enrichment of each regulator within the gene expression signature (GES) calculated between PD subgroups and healthy controls. Compared to the single-sample VIPER, the msVIPER integrated signal changes to compare differences between subgroups. The VIPER algorithm has been demonstrated to capture protein-level regulatory effects from transcriptomic data in many studies (Holland *et al*. 2020, Anglada-Girotto *et al*. 2025). Therefore, msVIPER in R with Discriminant Regulon Expression Analysis (DoRothEA) as a regulon source was employed in this study to infer the relative activity of TFs across PD subgroups. Significant TFs for each subgroup were listed in Supplementary Table S3.

Meanwhile, this study performed differential expression analysis of kinase-encoding genes to identify regulatory kinases exhibiting significant expression changes in each subgroup, contrasting to HC. The commonly used DESeq2 in R was adopted in this study. DESeq2 provides robust normalization and statistically principled modeling of RNA-seq count data, making it well suited for analyses involving heterogeneous samples and longitudinal cohort data in PPMI (Liu *et al*. 2021). Genes were considered statistically significantly differentially expressed at an adjusted *p*-value <0.05. To capture biologically relevant yet modest expression changes, subgroup-specific $\left|{\;\text{log}\;}_{2}\text{FC}\right|$ thresholds were applied within the range of 0.3 to 0.75, depending on the distribution of kinase-encoding gene expression in each subgroup. Specifically, the $\left|{\;\text{log}\;}_{2}\text{FC}\right|$ thresholds for the Sporadic PD, LRRK2, and GBA subgroups within the PD cohort were set at 0.30, 0.40, and 0.50, respectively, while the $\left|{\;\text{log}\;}_{2}\text{FC}\right|$ threshold for the SWEDD subgroup was 0.50. In the Prodromal cohort, the thresholds for the LRRK2, GBA, RBD, and Hyposmia subgroups were 0.75, 0.30, 0.30, and 0.35, respectively. Although a relatively low threshold of 0.3 was applied for some subgroups because few kinase-encoding genes would have been retained at higher cutoffs, this threshold was biologically justified, as kinases function catalytically and even minor alterations in their abundance can lead to amplified effects at the signaling level. A threshold of 0.3 has also been used in other studies, such as brain transcriptomics analyses (Alidadiani *et al*. 2025) and gene network and signaling pathway analyses in stroke (Mao *et al*. 2025). Additionally, downstream network analyses integrated multiple orthogonal sources of evidence, including VIPER-based TF activity inference and curated kinase–TF interactions from the SIGNOR 4.0 (Lo Surdo *et al*. 2026). As a result, even a relatively modest $\left|{\;\text{log}\;}_{2}\text{FC}\right|$ threshold can yield meaningful findings while retaining the top few most differentially expressed kinase-encoding genes for further analysis. The $\left|{\;\text{log}\;}_{2}\text{FC}\right|$ thresholds applied to each subgroup, along with the corresponding lists of differentially expressed kinases inferred from the differentially expressed genes, were summarized in Supplementary Table S3.

After identifying differentially expressed kinases, this study integrated these kinases with significant TFs and cross-referenced with SIGNOR 4.0 database to extract kinase–TF pairs where the kinase was significantly differentially expressed and the TF showed significant differential activity inferred by msVIPER. SIGNOR 4.0 provides comprehensive human kinases and substrates direct interactions, with regulation significance score and direction, namely up-regulation or down-regulation, included. The SIGNOR-supported kinase–TF pairs were then subjected to functional pathway enrichment analysis to characterize their biological context. Enrichment analysis was based on GO, KEGG, and Reactome databases, implemented in the R package. After that, PD subgroup-specific kinase–TF regulatory networks based on SIGNOR-supported kinase–TF pairs were constructed. Network nodes represented kinases or TFs, while edges denoted directional regulatory relationships (activation or inhibition) annotated by SIGNOR 4.0. Network construction and visualization were both performed in R. Finally, all subgroup-specific kinase–TF networks were integrated into a comprehensive network to capture both shared and distinct signaling alterations across PD subgroups. This integrative strategy moved beyond differential expression alone to reveal upstream signaling mechanisms that might selectively drive transcriptional changes in each PD subgroup. The interaction network was then interpreted with functional enrichment results to identify consistent biological processes and potential regulatory modules behind signaling alterations.

## Results

### TF activity and kinase expression

**Figure 2:**
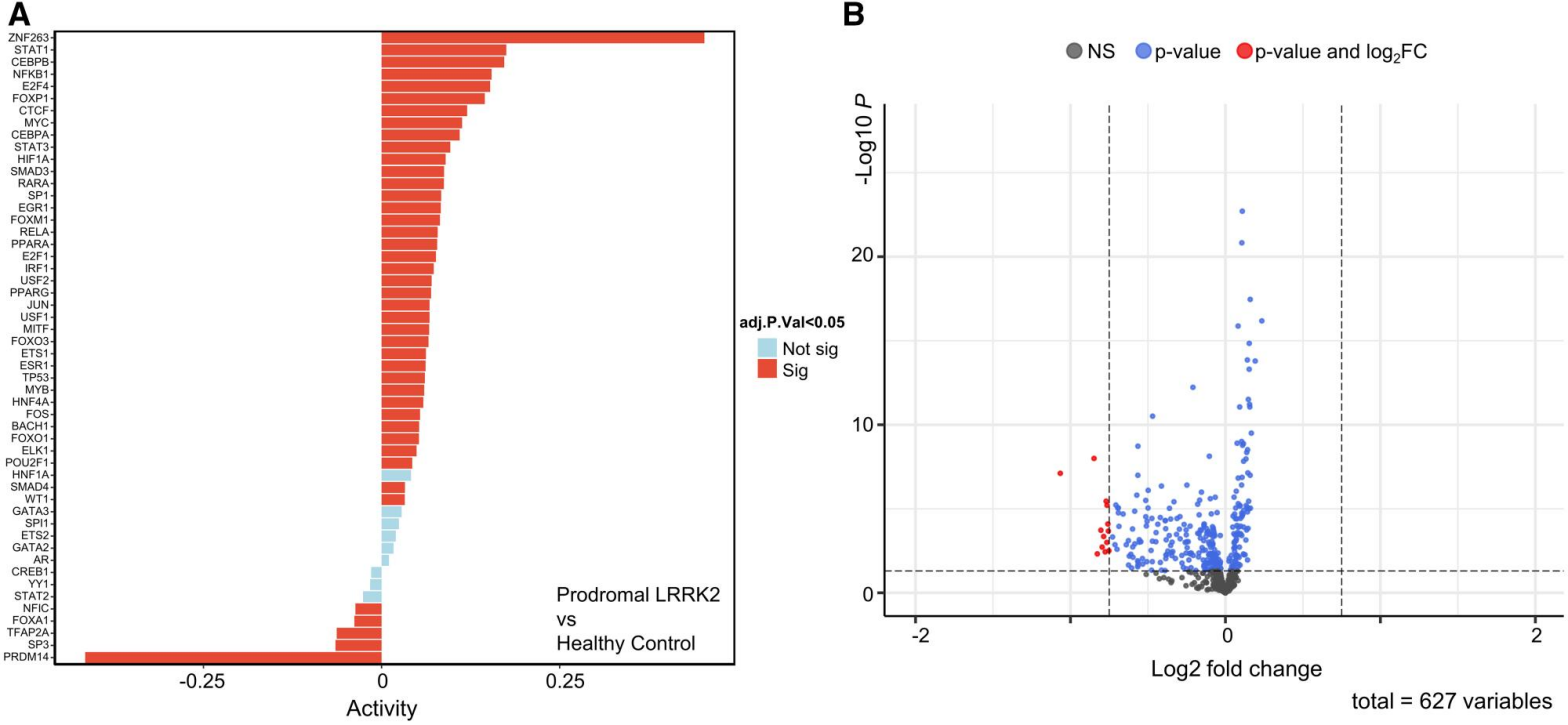
RNAseq analysis results for Prodromal_LRRK2 subgroup contrasting to healthy control subgroup. (A) The msVIPER analysis result represents the significant TFs (marked in red), and the adjusted *P*-value (adj.P.Val) was set to be less than 0.05. (B) DESeq2 enhanced volcano plot displays the differentially expressed kinases, the |log2FC| cutoff was set at 0.75, and the significance threshold, adjusted *P*-value, was set at 0.05.

As mentioned in the previous section, TF activities were assessed with msVIPER, and differential expression was performed to evaluate the expression level of kinases. The TF activities estimate the functional regulatory state of each TF based on the expression patterns of its target genes. Positive activity values indicate transcriptional activation, negative values indicate repression, and the magnitude reflects the strength of the regulatory change. These scores are normalized for cross-subgroup comparisons (Alvarez *et al*. 2016). Compared to the healthy control subgroup, TFs with significantly altered activities in the Prodromal_LRRK2 subgroup are presented in Fig. 2A. Multiple TFs exhibit significant activity, while only a few kinases show significant downstream regulatory expression differences. As mentioned previously, different subgroups may have used different values within the $\left|{\;\text{log}\;}_{2}\text{FC}\right|$ thresholds we applied. The $\left|{\;\text{log}\;}_{2}\text{FC}\right|$ value used by each subgroup, along with significant TFs and DE kinases, are listed in Supplementary Table S3.

The enhanced volcano plot demonstrating the relationship between statistical significance and the magnitude of fold change for genes is shown in Fig. 2B. Since kinases were required for further analysis, only genes encoding kinases were included in Fig. 2B to reduce noise. The results for the other seven subgroups can be found in Supplementary Fig. S1.

**Figure 3:**
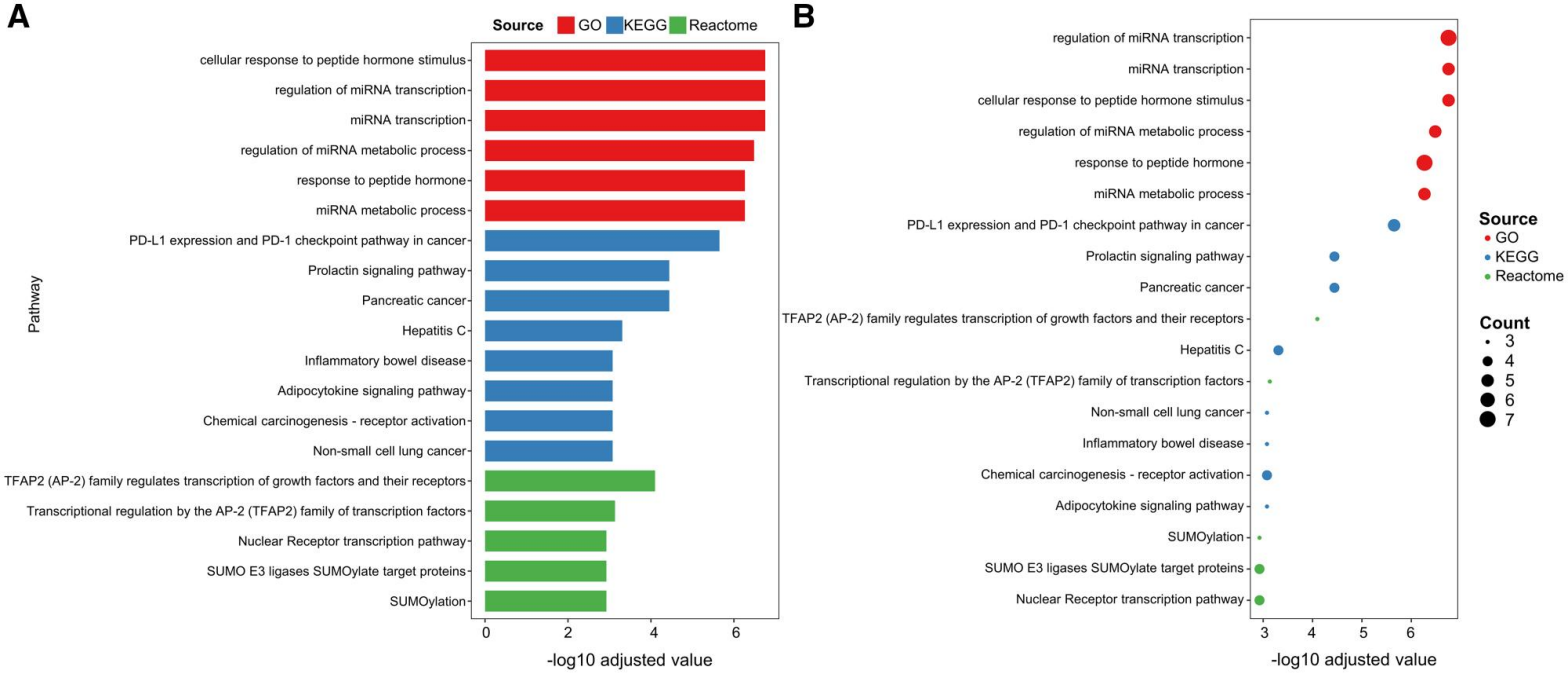
Pathway enrichment results for Prodromal_LRRK2 subgroup, with three colors representing three databases: GO, KEGG, and Reactome. The top five most significant pathways are displayed via (A) bar plot and (B) Unified dotplot, where dot size indicates gene counts, and the significance threshold, adjusted *P*-value, for each database was set at 0.05.

After kinases with significantly differential expression levels and TFs with significantly altered activities were obtained, SIGNOR 4.0 was employed in this study to retrieve potential kinase–TF phosphorylation signals across PD subgroups. The resulting list of phosphorylation interactions reported in SIGNOR 4.0 among these kinases and TFs is presented in Table 1. Pathway enrichment analysis was performed for each PD subgroup to find enriched functional pathways. The bar plot and unified dotplot for pathways sourced from GO, KEGG, and Reactome for the Prodromal_LRRK2 are presented in Fig. 3A and B, respectively. In the figure, only the top five pathways from each database are displayed to emphasize the most representative findings. The corresponding plots for other PD subgroups are provided in Supplementary Fig. S2.

**Table 1:** Summary of differentially expressed kinases, significantly altered TFs, and kinase–TF pairs with phosphorylation evidence derived from the SIGNOR 4.0 database, including a complete list of their associated interactions across PD subgroups.

PPMI cohorts	Subgroups	No. of DE kinases	No. of significant TFs (adjusted *P* value < 0.05)	List of Kinases and TFs with interactions in SIGNOR 4.0	Phosphorylation interaction in SIGNOR 4.0
PD	Sporadic PD	6	14	Kinase: EGFR, ALK TF: ESR1, STAT3	EGFR→ESR1; EGFR→STAT3; ALK→STAT3
	LRRK2	12	40	Kinase: PAK5, PRKAA2, PBK, ALK TF: RELA, HNF4A, JUN, STAT3	PAK5→RELA; PRKAA2→HNF4A; PBK→JUN; ALK→STAT3
	GBA	68	7	Kinase: CAMK2A, EPHA3, MAPK10, PAK6 TF: ETS2, AR, TP53	CAMK2A→ETS2; EPHA3→AR; MAPK10→TP53; PAK6→AR
SWEDD	SWEDD	29	2	Kinase: EGFR, FGFR3, ALK TF: STAT3	EGFR→STAT3; FGFR3→STAT3; ALK→STAT3
Prodromal	LRRK2	13	43	Kinase: EGFR, PAK5, PRKAA2, ALK TF: ESR1, STAT3, RELA, TFAP2A, STAT1, PPARG, HNF4A	EGFR→ESR1; EGFR→STAT3; PAK5→RELA; PRKAA2→TFAP2A; EGFR→STAT1; EGFR→PPARG; PRKAA2→HNF4A; ALK→STAT3
	GBA	29	20	Kinase: AURKB, TNK2, EPHA3 TF: TP53, AR, STAT3, STAT1	AURKB→TP53; TNK2→AR; TNK2→STAT3; TNK2→STAT1; EPHA3→AR
	RBD	33	20	Kinase: CDK1, VRK1, JAK1, PBK, TTK TF: TP53, JUN, STAT3	CDK1→TP53; VRK1→JUN; JAK3→STAT3; PBK→JUN; VRK1→TP53; TTK→TP53
	Hyposmia	19	28	Kinase: PRKCD, MAPK10 TF: CEBPA, TP53, STAT3	PRKCD→CEBPA; PRKCD→TP53; MAPK10→TP53; PRKCD→STAT3

### Kinase–TF interactions and pathways

**Figure 4:**
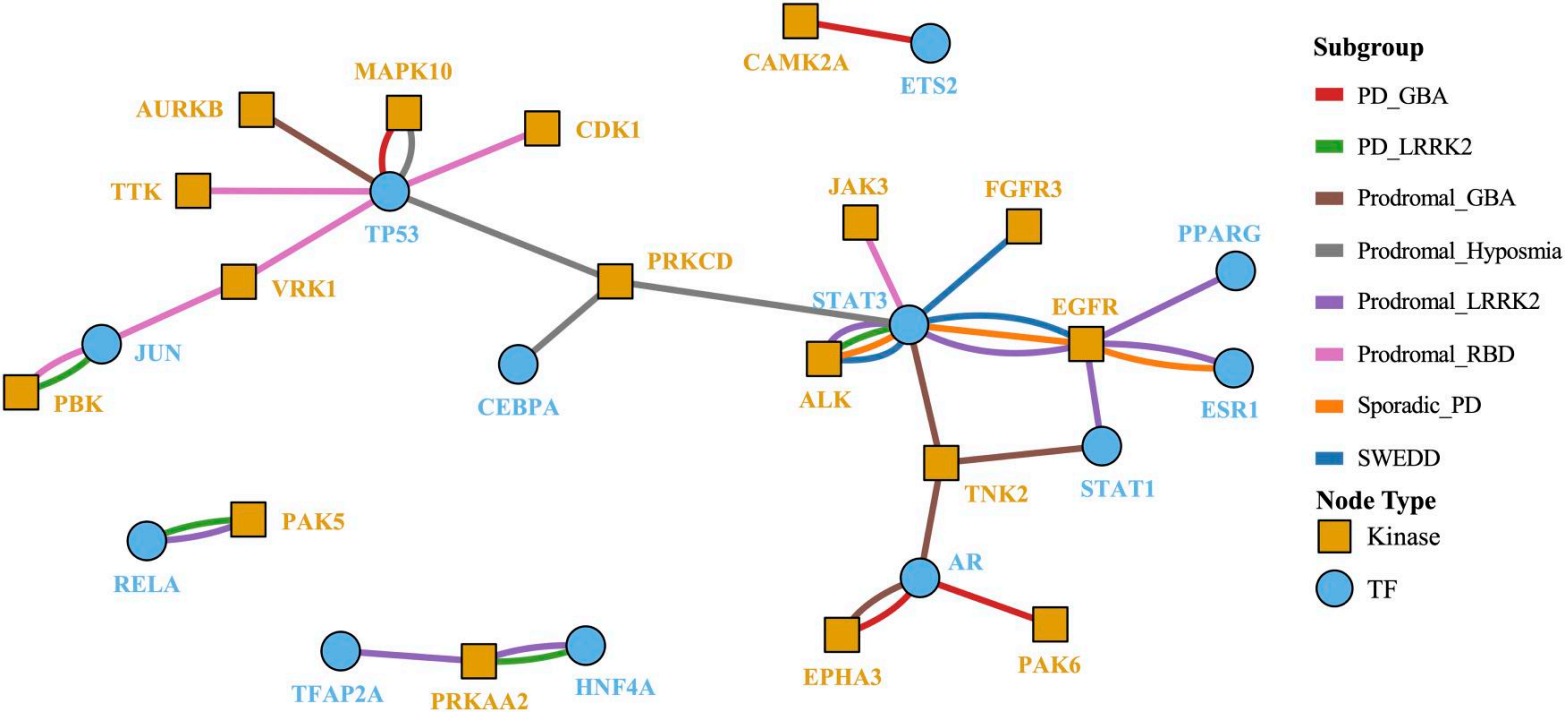
Overall kinase–TF interaction networks across PD subgroups, demonstrating both convergence and heterogeneity across subgroups.

The phosphorylation-based regulatory interactions between differentially expressed kinases and TFs with significantly altered activity in each subgroup are summarized in an integrated signaling network, as demonstrated in Fig. 4. All kinase–TF interactions were curated based on direct phosphorylation evidence from SIGNOR 4.0. As shown in Fig. 4, epidermal growth factor receptor (EGFR) to STAT3 represents a shared signaling axis across multiple early and classical PD subgroups, including SWEDD, sporadic PD, and Prodromal_LRRK2. Another common axis is ALK→STAT3, which appears in the SWEDD, sporadic PD, PD_LRRK2, Prodromal_LRRK2 subgroups. More details for each subgroup, including the total number of differentially expressed kinases, TFs with significantly altered activities, the interacting kinases and TFs, and the interactions supported by SIGNOR 4.0, are summarized in Table 2. The complete list of these kinases and TFs is provided in Supplementary Table S3.

**Table 2:** Summary of each kinase–TF interaction inferred from this study and its representative potentially PD-relevant molecular mechanism and pathways from the enrichment results across subgroups.

PD subgroup	Kinase → TF	Representative PD-relevant molecular mechanism	Representative pathways	Ref
SWEDD/Sporadic PD/PD LRRK2/Prodromal LRRK2	ALK → STAT3	Neuroinflammation	Signaling by ALK	Hamada *et al.* (2021)
SWEDD/Sporadic PD/Prodromal LRRK2	EGFR → STAT3	Dopaminergic neuron survival	JAK-STAT signaling pathway	Jin *et al.* (2020)
SWEDD	FGFR3 → STAT3	Neuroinflammation/neuron survival	Cell surface receptor signaling pathway via JAK-STAT	Lee *et al.* (2014)
Sporadic PD/Prodromal LRRK2	EGFR → ESR1	Neuroprotection/dopaminergic neuron survival	Neurotrophin signaling pathway	Romano and Bucci (2020)
PD LRRK2/Prodromal LRRK2	PAK5 → RELA (NF-κB)	Neuroinflammation and survival signaling	Ras signaling pathway	Zhang *et al.* (2017)
PD LRRK2/Prodromal LRRK2	PRKAA2 → HNF4A	Mitochondrial function/energy metabolism	Response to carbohydrate	Santiago and Potashkin (2015)
PD LRRK2/Prodromal RBD	PBK → JUN (AP-1)	Oxidative stress/neuroinflammation	MAPK signaling pathway	Li *et al.* (2016)
PD GBA/Prodromal LRRK2	MAPK10 (JNK3) → TP53	Apoptosis/mitochondrial stress	JNK3/p53 stress pathway	Nakano *et al.* (2020)
PD GBA/Prodromal LRRK2	EPHA3 → AR	Axonal transport/neuron connectivity	regulation of protein localization to plasma membrane	Diao *et al.* (2018)
PD GBA	CAMK2A → ETS2	Neuron survival/synaptic plasticity	Neurotrophin signaling pathway	Jack *et al.* (2009)
PD GBA	PAK6 → AR	Dopaminergic neuron function/survival	Insulin-like growth factor receptor signaling pathway	Iannotta *et al.* (2024)
Prodromal LRRK2	PRKAA2 → TFAP2A	Oxidative stress response	KEAP1-NFE2L2 pathway	Ma *et al.* (2017)
Prodromal LRRK2	EGFR → STAT1	Neuroinflammation	JAK-STAT signaling pathway	Ferluga *et al.* (2020)
Prodromal LRRK2	EGFR → PPARG	Cell survival/proliferation signaling	Epithelial cell proliferation	Xu *et al.* (2016)
Prodromal GBA	AURKB → TP53	DNA damage response/apoptosis	Cell cycle checkpoint signaling	Marima *et al.* (2021)
Prodromal GBA	TNK2 → AR	Signal transduction/neuronal signaling	Positive regulation of phosphorylation	Mahajan *et al.* (2017)
Prodromal GBA	TNK2 → STAT3	Neuroinflammation	Peptidyl-tyrosine phosphorylation	Mahendrarajah *et al.* (2017)
Prodromal GBA	TNK2 → STAT1	Immune response/neuroinflammation	Regulation of clathrin-dependent endocytosis	Mahendrarajah *et al.* (2017)
Prodromal RBD	CDK1 → TP53	Apoptosis/cell cycle dysregulation	p53 signaling pathway	Wang *et al.* (2023)
Prodromal RBD	VRK1 → JUN	Stress response/neuroinflammation	MAPK family signaling cascades	Sevilla *et al.* (2004)
Prodromal RBD	JAK3 → STAT3	Metabolic dysfunction/neuron survival	JAK-STAT signaling pathway	Krolopp *et al.* (2016)
Prodromal RBD	VRK1 → TP53	DNA damage response/apoptosis	p53 signaling pathway	Campillo-Marcos *et al.* (2021)
Prodromal RBD	TTK → TP53	DNA damage response/apoptosis	Mitotic cell cycle checkpoint signaling	Huang *et al.* (2009)
Prodromal Hyposmia	PRKCD → CEBPA	Neuroinflammation/apoptosis	Regulation of inflammatory response	Zhao *et al.* (2009)
Prodromal Hyposmia	PRKCD → TP53	Apoptosis/cell death	Apoptosis	Grison *et al.* (2011)
Prodromal Hyposmia	PRKCD → STAT3	Neuroinflammation/survival	Negative regulation of inflammatory response	Panicker *et al.* (2015)

Supporting literature references are provided for interaction evidence reported in PD or other disease contexts.

**Figure 5:**
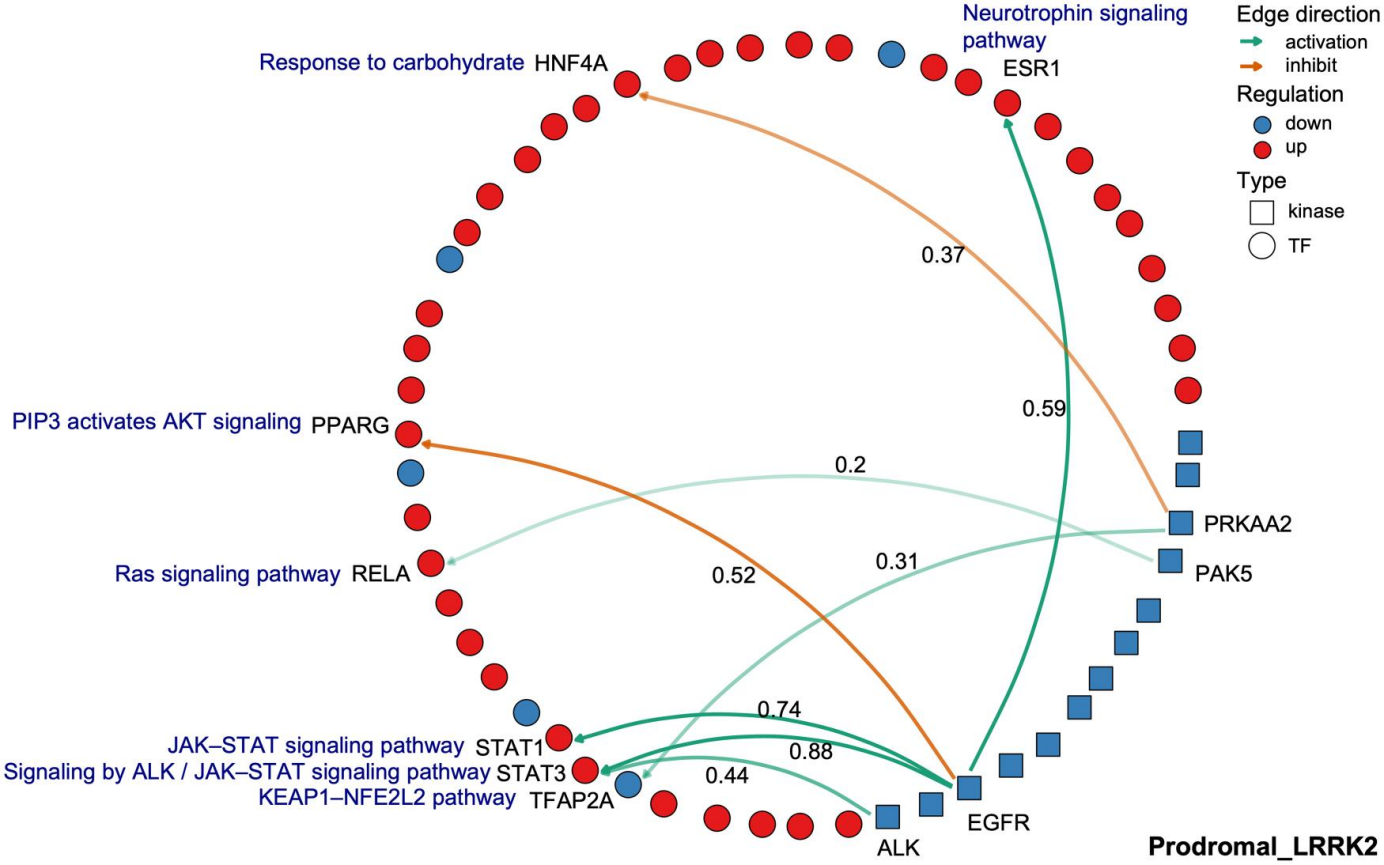
Kinase–TF regulatory network and associated functional pathways in the Prodromal_LRRK2 subgroup. The network illustrates inferred signaling interactions from kinases to their targeted TFs, alongside representative pathways potentially relevant to PD pathology. Scores next edges indicate interaction confidence scores obtained from the SIGNOR 4.0 database.

Since pathway enrichment analysis was performed for each subgroup using the interacting kinases and TFs, as demonstrated in the previous subsection, the kinase–TF interactions and their representative pathways are illustrated in Fig. 5. Figure 5 shows the results for the Prodromal_LRRK2 subgroup, incorporating the Integrated Network and Dynamical Reasoning Assembler (INDRA) score (Lo Surdo *et al*. 2026), which represents the confidence level of each kinase–TF interaction to prioritize curation. The complete list of kinase–TF interactions for all subgroups, along with their representative pathways, is provided in Table 2. These pathways have been reported in the literature to be associated with the corresponding kinase–TF phosphorylation interactions, as evidenced by studies in PD or other diseases.

## Conclusion and discussion

The novel integrative regulatory modeling strategy in this study combined msVIPER-inferred TF activity with DESeq2-identified differentially expressed kinases, and incorporated curated phosphorylation relationships from SIGNOR 4.0 to infer potential subgroup-specific kinase–TF signaling networks. This study’s framework led to the identification of both convergence and heterogeneity across various PD subgroups, providing novel insights into stratified subgroup-dependent molecular pathophysiology and can inform the development of more precise therapeutic interventions for treating various PD subgroups as a result.

As shown in Table 1, variation in the number of significant kinases and TFs across PD subgroups can be observed. Although different $\left|{\;\text{log}\;}_{2}\text{FC}\right|$ thresholds applied across subgroups during differential expression analysis might partially explain this difference, these differences may be further influenced by subgroup-specific heterogeneity, such as cellular composition, disease stage, and the extent of network rewiring, leading to the variation in the numbers of significantly altered kinases and TFs. These results highlight that PD subgroups may demonstrate unique signaling landscapes. Therefore, subgroup-specific analyses should be performed when investigating kinase–TF regulatory mechanisms in PD. Using the sporadic PD subgroup as a representative case, we observed clear subgroup-specific regulatory imbalances at the transcriptional level. msVIPER-inferred results revealed MYC exhibited the most significant decrease in inferred activity, which can be observed from Supplementary Fig. S1, consistent with evidence that MYC proto-oncogene, bHLH transcription factor (MYC) was the major TF in modulating the hub genes related to PD in their study (Odumpatta and Arumugam 2021). In contrast, the increased activities of STAT3, Spi-1 proto-oncogene (SPI1), and estrogen receptor 1 (ESR1) highlight enhanced neuro-inflammatory and immune-associated transcriptional programs. Increased activity of regulators such as tumor protein P53 (TP53) and ETS proto-oncogene 2, transcription factor (ETS2) further suggests a shift toward stress response and survival pathways (Mitra *et al*. 2025, Abate *et al*. 2020). Some TFs observed in other subgroups were also closely related to PD. For instance, the NF-κB (RELA proto-oncogene, NF-kB subunit), which can be observed in the PD_LRRK2 and Prodromal_LRRK2 stratified subgroups, regulates the expression of nearly 150 genes that play a statistically significant role in protective immunity and pro-inflammatory pathways (Rai *et al*. 2021).

The dysregulation of specific kinases is critical in the pathophysiology of PD. For instance, several kinases were found to be downregulated in the Prodromal_LRRK2 subgroup, as shown in Fig. 2 and Table S2. Among these, neurotrophic receptor tyrosine kinase 3 (NTRK3) has been reported to participate in neurotrophic signaling and neuronal survival (Braskie *et al*. 2013). In addition, the catalytic subunit protein kinase AMP-activated catalytic subunit alpha 2 (PRKAA2) of AMP-activated protein kinase (AMPK) has been associated with neurodegenerative disorders (Wu *et al*. 2025). These observations suggest that the differentially expressed kinases identified here may play critical roles in PD-related signaling pathways. Furthermore, integrating the differentially expressed kinases with TFs showing significantly altered activities can help interpret the pathophysiological impact of kinase–TF regulatory networks underpinning PD.

As demonstrated in Fig. 4, the inferred regulatory network revealed both convergence and heterogeneity across PD subgroups. A recurring feature was the activation of STAT family TFs downstream of receptor tyrosine kinases (RTKs, such as EGFR and anaplastic lymphoma kinase (ALK)) and non-receptor tyrosine kinases (NRTKs, such as tyrosine kinase non-receptor 2 (TNK2)/activated cdc42-associated Kinase 1 (ACK1), protein kinase C delta (PRKCD), and Janus kinase 3 (JAK3)), suggesting a common pro-survival/inflammatory axis in several clinical subgroups, namely SWEDD, sporadic PD, and Prodromal_LRRK2 subgroup. EGFR and ALK are established upstream activators of STAT3 in multiple diseases (Pal *et al*. 2016, Hamada *et al*. 2021), including PD (Jin *et al*. 2020). Despite some shared interactions, subgroup-specific networks revealed some biological heterogeneity. In the PD_LRRK2 subgroup, p21-activated kinase 5 (PAK5) to RELA and PRKAA2 to hepatocyte nuclear factor 4 alpha (HNF4A) might suggest a coupling of Ras and AMPK-mediated signaling that integrates energy metabolism with NF-κB-driven neuroinflammation (Zhang *et al*. 2017, Santiago and Potashkin 2015, Hong *et al*. 2003). These interactions align with known mitochondrial dysfunction and immune activation in LRRK2 carriers (Wallings *et al*. 2020). GBA-associated subgroups displayed strong engagement of EPH receptor A3 (EPHA3) to androgen receptor (AR) and calcium/calmodulin-dependent protein kinase II alpha (CAMK2A) to ETS2 axes tied to vesicular trafficking, synaptic maintenance, and dopaminergic neuron survival (Diao *et al*. 2018, Jack *et al*. 2009). The inclusion of AR regulation, also observed through TNK2→AR in Prodromal_GBA, might suggest a possible role for hormone-mediated mechanisms in GBA-related pathophysiology. The prominence of apoptosis- and DNA damage-related signaling, such as Aurora kinase B (AURKB) to TP53 (Marima *et al*. 2021) and TTK protein kinase (TTK) to TP53 (Huang *et al*. 2009), in Prodromal_GBA and Prodromal_RBD aligns with increased conversion risk and more aggressive neurodegenerative trajectories. Prodromal subgroups also exhibited early activation of inflammatory and stress pathways, as demonstrated by EGFR→STAT1, PRKCD→STAT3, and PRKCD→TP53 in Prodromal_LRRK2 and Prodromal_hyposmia (Ferluga *et al*. 2020, Grison *et al*. 2011, Panicker *et al*. 2015, Onodera *et al*. 2018), which suggests that inflammation-induced cell death may precede motor symptom onset, potentially offering a signaling-based window for early therapeutic intervention.

It should be noted that some kinase–TF interactions inferred from this study are well established in neurodegenerative disorder studies in the literature, for instance, the regulatory links of EGFR→STAT3 (Jin *et al*. 2020), PRKCD→TP53 (Grison *et al*. 2011), PDZ-binding kinase (PBK) to Jun proto-oncogene, activator protein 1 (JUN/AP-1) (Zhang *et al*. 2025, Li *et al*. 2016). Common downstream pathways such as mitogen-activated protein kinase (MAPK) and JAK-STAT, which have important roles in inflammation, have been proposed as potential therapeutic targets in PD (Qin *et al*. 2016). Although some kinase–TF interactions were not directly reported in the PD pathogenesis, all these interactions, as reported in Table 2 have direct support in other diseases such as leukemic cells (Zhao *et al*. 2009), skeletal muscle cells (Krolopp *et al*. 2016), cancer cells (Xu *et al*. 2016, Campillo-Marcos *et al*. 2021), supporting the biological plausibility of these inferred links. It also highlights the significance of this study, which shows that the inferred kinase–TF interactions not reported so far in the PD pathology might serve as potential effective targets in the intervention and treatment of PD disease. For instance, Odumpatta and Arumugam [2021] suggested that the PKC family can be a significant druggable target for PD, and PRKCD in the Prodromal_hyposmia subgroup is a member of the PKC in the regulatory network of this study, demonstrating the therapeutic potential of kinases and TFs retained from this study.

Significantly, the comparison of potential regulatory networks across subgroups might contribute to understanding the progression of PD. For instance, comparison of Prodromal_LRRK2 and PD_LRRK2 shows that, although they shared several kinase–TFinteractions, Prodromal_LRRK2 demonstrated stronger EGFR/STAT and hormone receptor links, as shown in EGFR to nuclear hormone receptors ESR1 and peroxisome proliferator-activated receptor gamma (PPARG). This pattern suggests that RTK-hormone receptor crosstalk may be an upstream event in PD development for individuals with LRRK2 mutations, consistent with the role of RTK-mediated signaling, growth factors, and related downstream molecules in neuronal function and development (Sengupta *et al*. 2024).

Considering the present findings, this approach has implications for uncovering disease-specific and shared kinase–TF regulatory mechanisms in other central nervous system (CNS) disorders. For example, neurodegenerative diseases such as multiple sclerosis (MS) and Alzheimer’s disease also involve complex signaling perturbations and inflammatory processes. Applying the method to these conditions could help clarify signaling pathways that contribute to the inflammatory processes and synaptic dysfunction, or processes that lead to neuronal death, with the potential to reveal other regulatory patterns. Such insights could explain the reasons for differential effects on certain neuronal structures or brain regions in different diseases and could help in identifying molecular biomarkers for early detection or disease progression.

In addition, the same framework can be applied in cancer research, where dysregulation of transcriptional control and kinase signaling is a hallmark of tumorigenesis. Understanding the kinase–TF regulatory pathways in different cancers could increase our understanding of the conserved oncogenic pathways (e.g. EGFR→STAT3, MAPK→MYC) and signaling mechanisms that impact the heterogeneity in tumours, drug resistance, and metastasis. Integration of TF activity with kinase alterations could help in identifying novel therapeutic targets and precision medicine strategies tailored to the patient’s needs. Overall, extending this workflow to other disorders can deepen our understanding of disease mechanisms and speed up the development of more targeted and effective treatments.

Despite the significance of the subgroup-specific kinase–TF regulatory network and the integrative workflow of this study, there are some limitations. First, kinase–TF interactions were inferred based on curated kinase-substrate information from SIGNOR 4.0, and reliance on it may bias well-characterized pathways and overlook novel or understudied regulatory events. This constraint may narrow the scope of detectable kinases and TFs, particularly given that a substantial fraction of human kinase-substrate relationships remain unvalidated. Nevertheless, this study selected the top differentially expressed kinases and TFs with significantly altered activities to reveal potential kinase–TF regulatory networks across PD subgroups, and such a bioinformatic-based workflow can narrow down the promising regulatory candidates for the intervention of PD. Second, kinase selection in this study was based solely on transcriptomic changes, while kinase activities based on phosphoproteomics would help to validate kinase activity. However, kinase activities were missing due to the lack of phosphoproteomics data in PPMI. As PPMI keeps updating, the combination of phosphoproteomics will be helpful in capturing the critical kinases across PD subgroups in the future. Third, experimental validation of the kinase–TF interactions across subgroups was missing. However, all kinase–TF interactions and their representative functional pathways obtained from this study were directly supported by PD or other disease studies in the literature. Additionally, all interacting kinases and TFs were based on differentially expressed levels and msVIPER inferred activity for the corresponding subgroup, respectively, ensuring these interacting networks’ relevance to the corresponding PD subgroup. An experimental validation will be crucial in the future to validate the results further. Additionally, this study has covered eight PD groups so far due to the small number of samples for other subgroups, such as individuals with parkin RBR E3 ubiquitin-protein ligase (PRKN) mutation, or patients who carry pathogenic mutations in more than one gene, such as both the LRRK2 and GBA genes, and both the PRKN and RBD genes. In the future, a more comprehensive analysis covering all these subgroups will provide a deeper understanding of PD pathology across subgroups. For instance, by considering transcriptomic, phosphoproteomic, and epigenomic data in the future, a more precise inference of kinase activity and its downstream regulatory effects across different PD subgroups will be possible. Additionally, drug repurposing analyses can be performed to advance kinase-centered therapeutic interventions for PD, which might lead to the identification of subgroup-specific therapeutic candidates with established safety profiles.

In conclusion, this study reveals that kinase–TF regulatory networks differ across PD subgroups, reflecting distinct regulatory mechanisms. Conserved signaling axes, such as ALK→STAT3 and EGFR→STAT3, highlight shared neuroinflammatory and survival pathways in some subgroups of PD, whereas subgroup-specific kinase–TF interactions reinforce the molecular heterogeneity of the disease. By integrating TF activity with upstream kinase alterations, the workflow developed here provides a strategy for identifying potential subgroup-tailored biomarkers and disease mechanisms. Actionable targets that could guide precision therapies and stratified clinical interventions are essential to improved diagnostics and interventions to slow or prevent PD.

## Supplementary Material

vbag059_Supplementary_Data

## Data Availability

Data used in the preparation of this article was obtained on [2025-06-20] from the Parkinson’s Progression Markers Initiative (PPMI) database (www.ppmi-info.org/access-dataspecimens/download-data), RRID: SCR_006431. For up-to-date information on the study, visit www.ppmi-info.org. The code supporting the methodologies and findings of this article is available on GitHub at https://github.com/xyzhou218/Kin_TF_net. PPMI—a public-private partnership—is funded by the Michael J. Fox Foundation for Parkinson’s Research and funding partners.
