# Supplementary material for: Stratified signaling network remodeling of kinase–transcription factors’ interactions in Parkinson’s disease
Source: Bioinform Adv. 2026 Feb 17;6(1):vbag059. doi: 10.1093/bioadv/vbag059 (PMC12955839; doi:10.1093/bioadv/vbag059)
Supplement: vbag059_Supplementary_Data [file vbag059_supplementary_data.zip › Figure S1_TF_Kin.pdf]

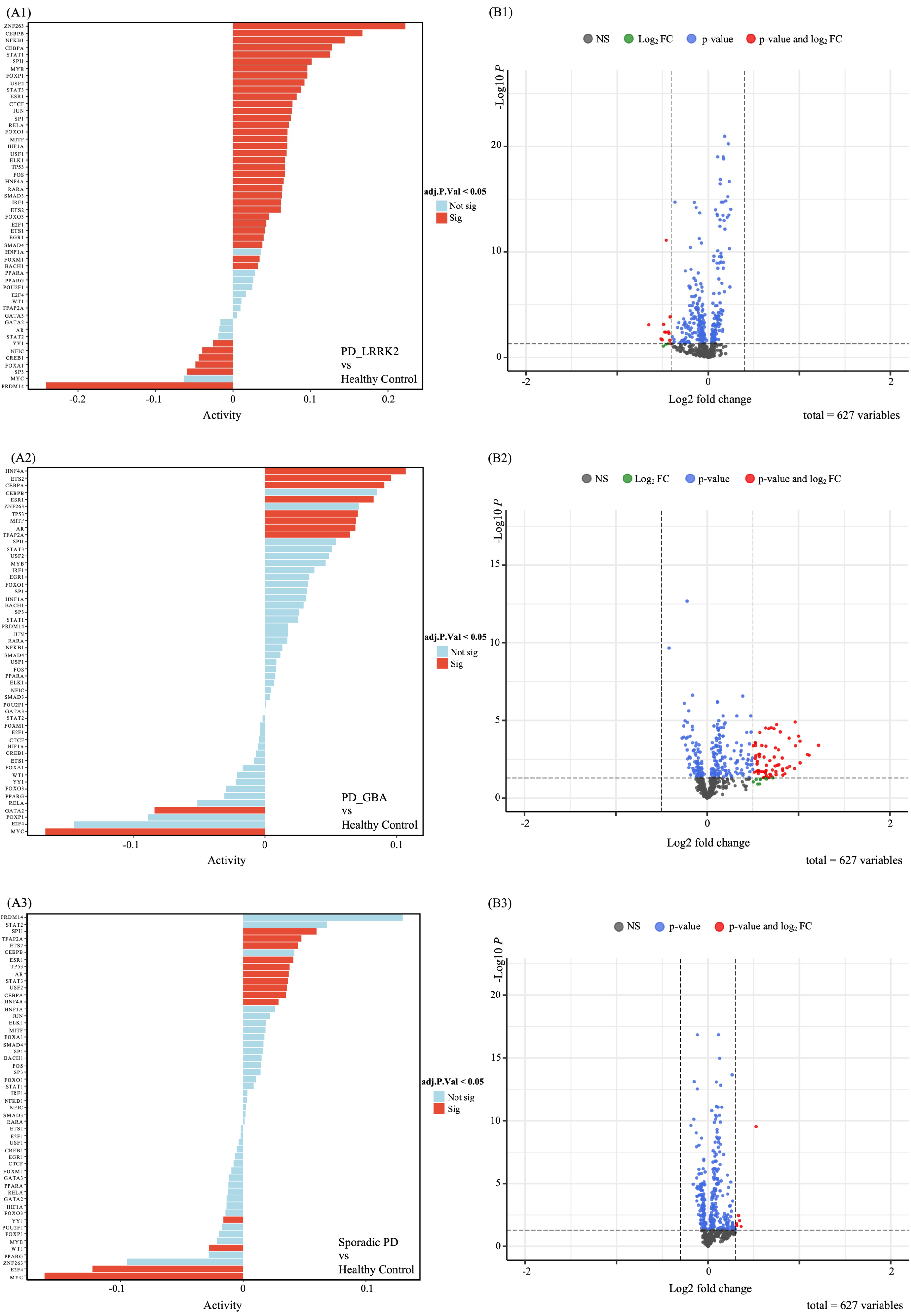

(A1)
ZNF263
CEBPB
NFKB1
CEBPA
STAT1
SPI1
MYB
FOXP1
USF2
STAT3
ESR1
CTCF
JUN
SP1
RELA
FOXO1
MITF
HIF1A
USF1
ELK1
TP53
FOS
HNF4A
RARA
SMAD3
IRF1
ETS2
FOXO3
E2F1
ETS1
EGR1
SMAD4
HNF1A
FOXM1
BACH1
PPARA
PPARG
POU2F1
E2F4
WT1
TFAP2A
GATA3
GATA2
AR
STAT2
YY1
NFIC
CREB1
FOXA1
SP3
MYC
PRDM14
PD_LRRK2
vs
Healthy Control
-0.2
-0.1
0
0.1
0.2
Activity
adj.P.Val < 0.05
Not sig
Sig
(B1)
NS
Log2 FC
p-value
p-value and log2 FC
-Log10 P
0
10
20
-2
0
2
Log2 fold change
total = 627 variables
(A2)
HNF4A
ETS2
CEBPA
CEBPB
ESR1
ZNF263
TP53
MITF
AR
TFAP2A
SPI1
STAT3
USF2
MYB
IRF1
EGR1
FOXO1
SP1
HNF1A
BACH1
SP3
STAT1
PRDM14
JUN
RARA
NFKB1
SMAD4
USF1
FOS
PPARA
ELK1
NFIC
SMAD3
POU2F1
GATA3
STAT2
FOXM1
E2F1
CTCF
HIF1A
CREB1
ETS1
FOXA1
WT1
YY1
FOXO3
PPARG
RELA
GATA2
FOXP1
E2F4
MYC
PD_GBA
vs
Healthy Control
-0.1
0
0.1
Activity
adj.P.Val < 0.05
Not sig
Sig
(B2)
NS
Log2 FC
p-value
p-value and log2 FC
-Log10 P
0
5
10
15
-2
0
2
Log2 fold change
total = 627 variables
(A3)
PRDM14
STAT2
SPI1
TFAP2A
ETS2
CEBPB
ESR1
TP53
AR
STAT3
USF2
CEBPA
HNF4A
HNF1A
JUN
ELK1
MITF
FOXA1
SMAD4
SP1
BACH1
FOS
SP3
FOXO1
STAT1
IRF1
NFKB1
NFIC
SMAD3
RARA
ETS1
E2F1
USF1
CREB1
EGR1
CTCF
FOXM1
GATA3
PPARA
RELA
GATA2
HIF1A
FOXO3
YY1
POU2F1
FOXP1
MYB
WT1
PPARG
ZNF263
E2F4
MYC
Sporadic PD
vs
Healthy Control
-0.1
0
0.1
Activity
adj.P.Val < 0.05
Not sig
Sig
(B3)
NS
p-value
p-value and log2 FC
-Log10 P
0
5
10
15
20
-2
0
2
Log2 fold change
total = 627 variables

(A4)

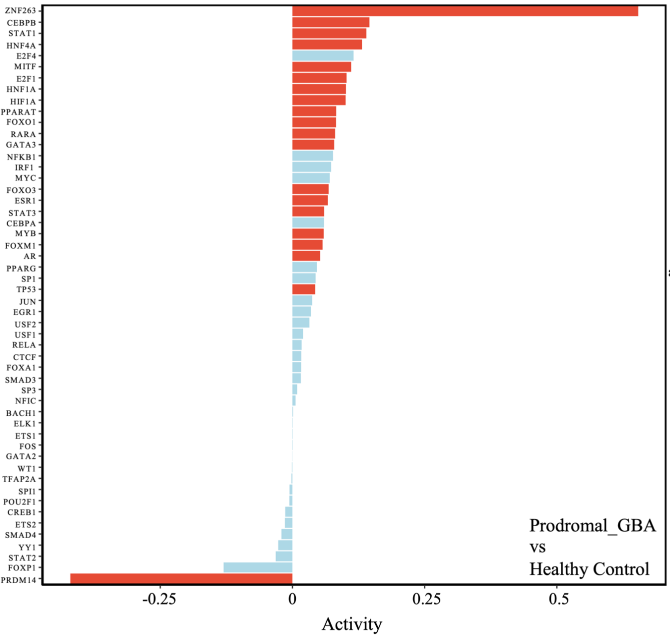

(B4)

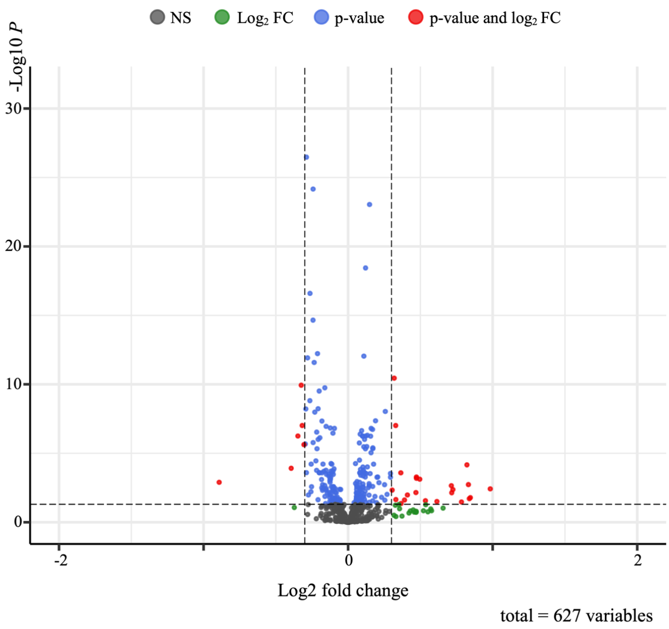

(A5)

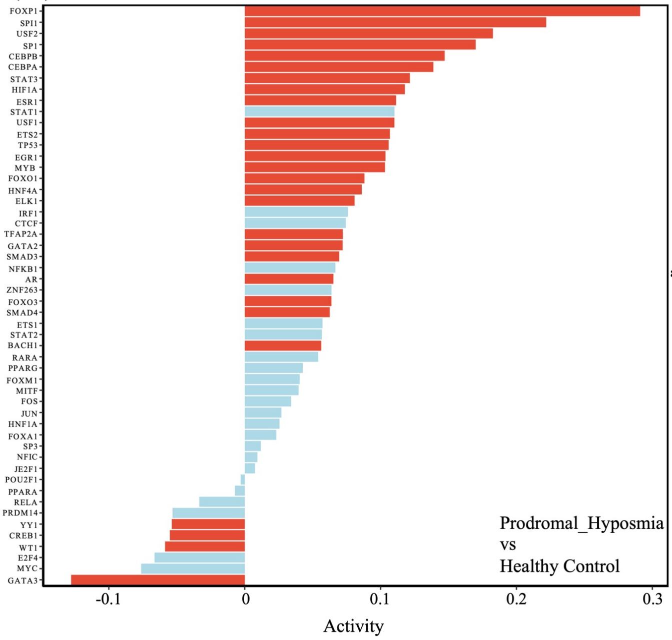

(B5)

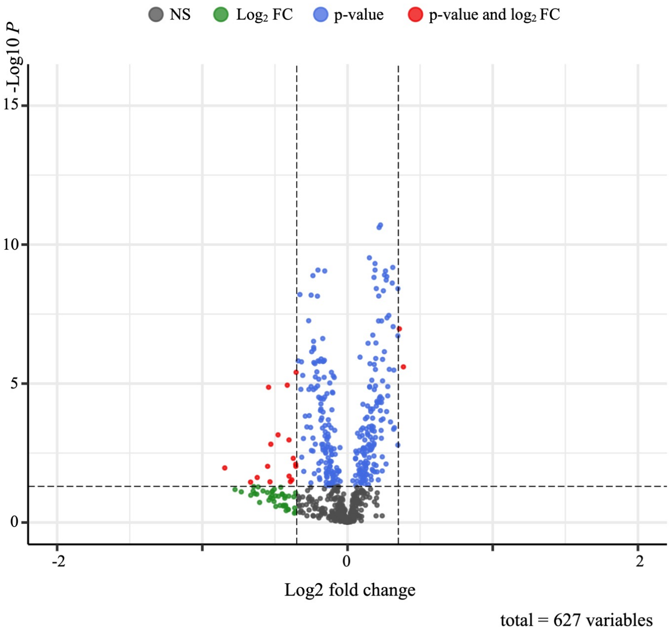

(A6)

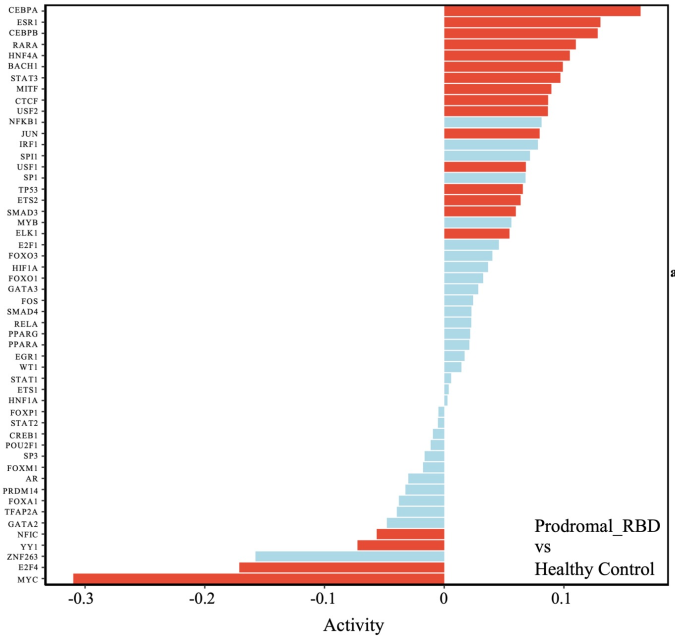

(B6)

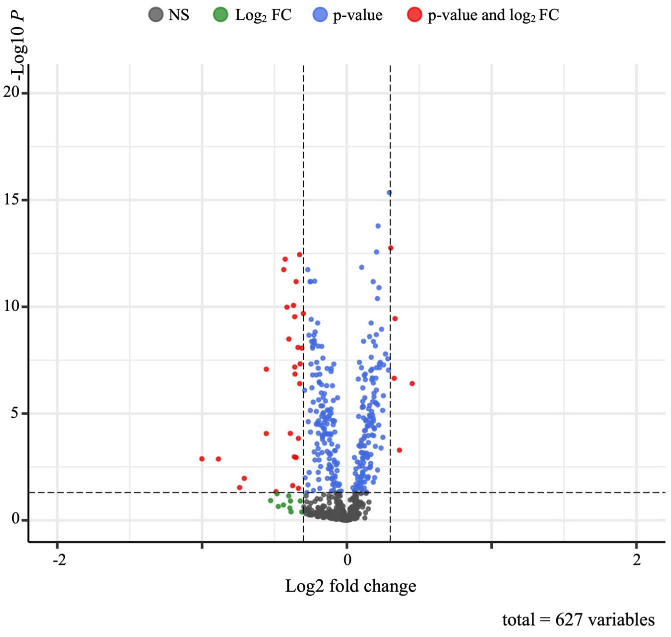

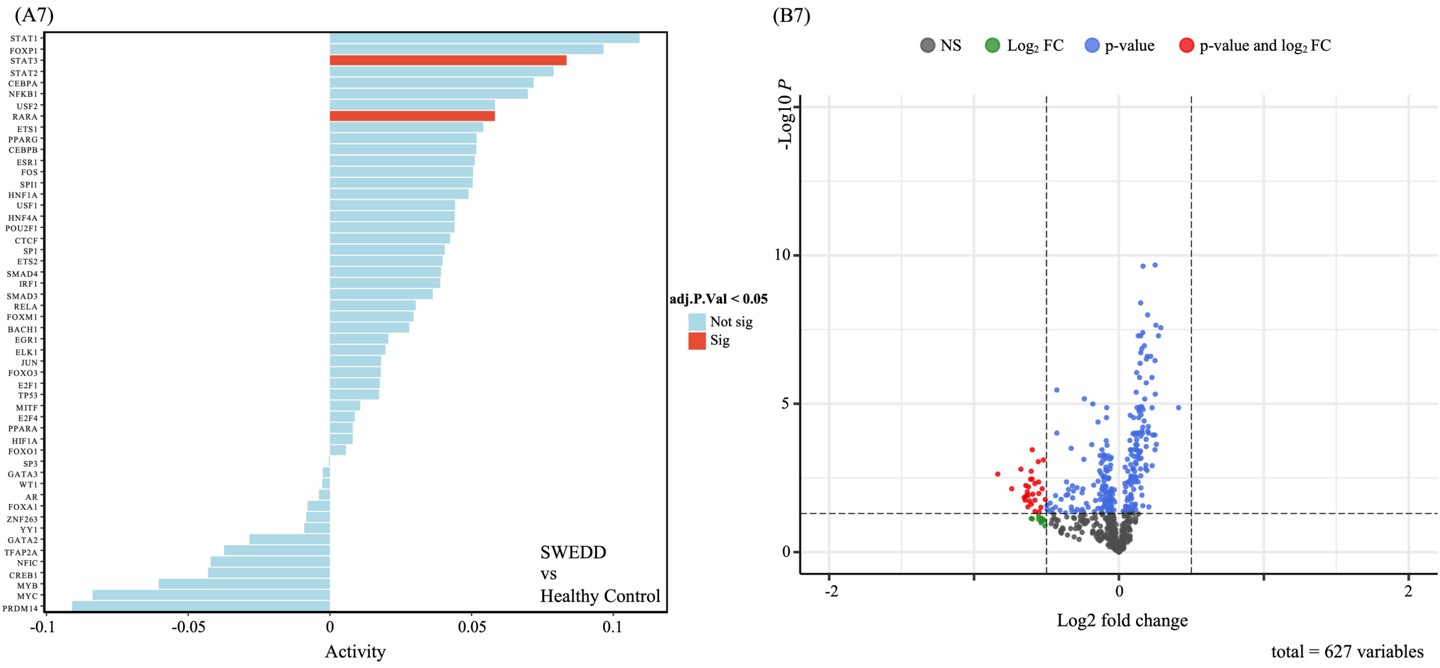

Supplementary Figure 1: RNAseq analysis results for PD_LRRK2, PD_GBA, sporadic_PD, prodromal_GBA, prodromal_hyposmia, prodromal_RBD and SWEDD subgroup contrasting to healthy control subgroup, respectively. (A) The msVIPER analysis result represents the significant TFs (marked in red), and the adjusted p-value (adj.P.Val) was set to be less than 0.05. (B) DESeq2 enhanced volcano plot displays the differentially expressed kinases, the |*log2FC*| cutoff was set at 0.75, and the significance threshold, adjusted p-value, was set at 0.05.
