# Supplementary material for: Stratified signaling network remodeling of kinase–transcription factors’ interactions in Parkinson’s disease
Source: Bioinform Adv. 2026 Feb 17;6(1):vbag059. doi: 10.1093/bioadv/vbag059 (PMC12955839; doi:10.1093/bioadv/vbag059)
Supplement: vbag059_Supplementary_Data [file vbag059_supplementary_data.zip › Figure S2_pathway.pdf]

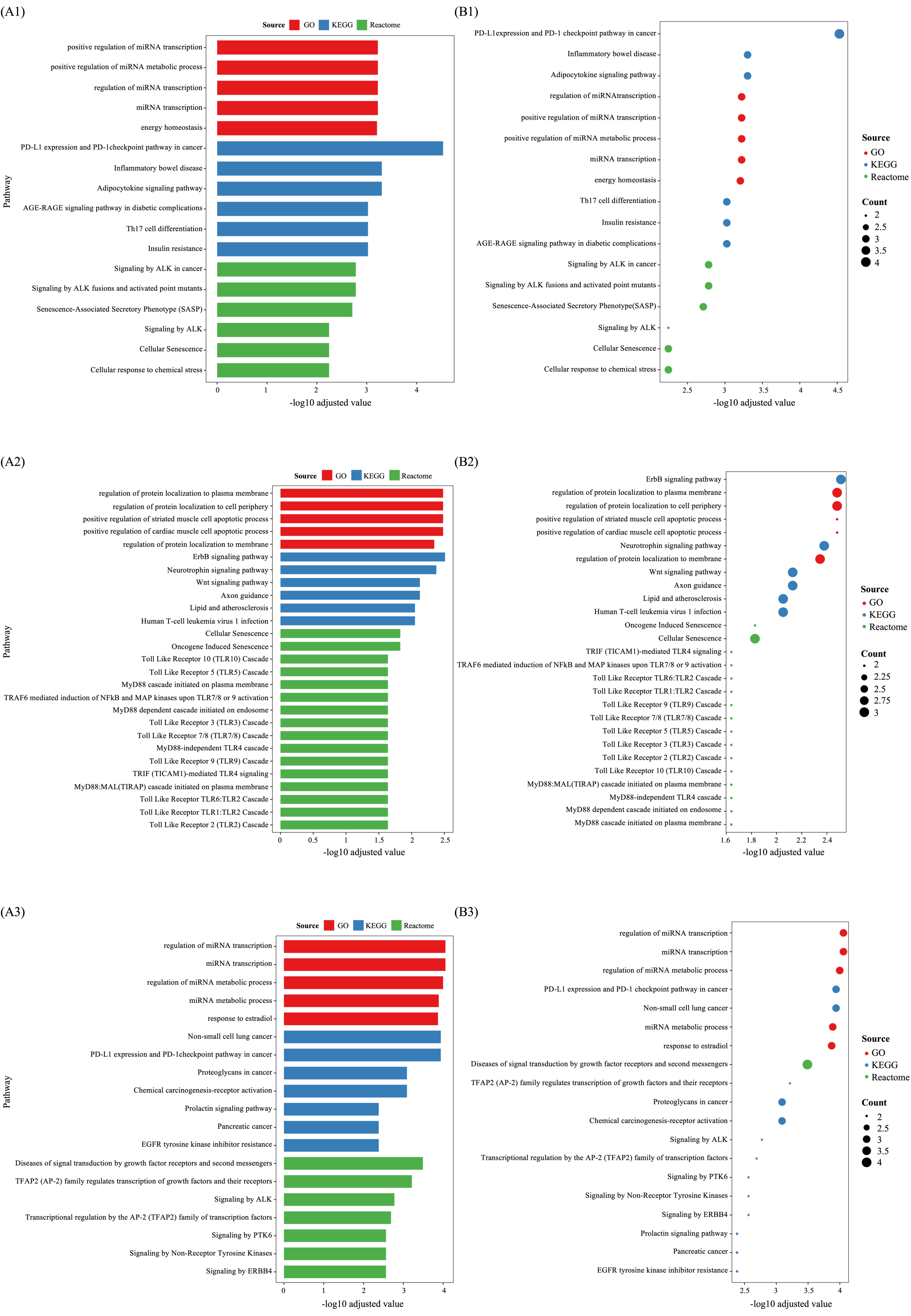
(A1)
Source GO KEGG Reactome
positive regulation of miRNA transcription
positive regulation of miRNA metabolic process
regulation of miRNA transcription
miRNA transcription
energy homeostasis
PD-L1 expression and PD-1checkpoint pathway in cancer
Inflammatory bowel disease
Adipocytokine signaling pathway
AGE-RAGE signaling pathway in diabetic complications
Th17 cell differentiation
Insulin resistance
Signaling by ALK in cancer
Signaling by ALK fusions and activated point mutants
Senescence-Associated Secretory Phenotype (SASP)
Signaling by ALK
Cellular Senescence
Cellular response to chemical stress
Pathway
0 1 2 3 4
-log10 adjusted value
(B1)
PD-L1expression and PD-1 checkpoint pathway in cancer
Inflammatory bowel disease
Adipocytokine signaling pathway
regulation of miRNAtranscription
positive regulation of miRNA transcription
positive regulation of miRNA metabolic process
miRNA transcription
energy homeostasis
Th17 cell differentiation
Insulin resistance
AGE-RAGE signaling pathway in diabetic complications
Signaling by ALK in cancer
Signaling by ALK fusions and activated point mutants
Senescence-Associated Secretory Phenotype(SASP)
Signaling by ALK
Cellular Senescence
Cellular response to chemical stress
2.5 3 3.5 4 4.5
-log10 adjusted value
Source
GO
KEGG
Reactome
Count
2
2.5
3
3.5
4
(A2)
Source GO KEGG Reactome
regulation of protein localization to plasma membrane
regulation of protein localization to cell periphery
positive regulation of striated muscle cell apoptotic process
positive regulation of cardiac muscle cell apoptotic process
regulation of protein localization to membrane
ErbB signaling pathway
Neurotrophin signaling pathway
Wnt signaling pathway
Axon guidance
Lipid and atherosclerosis
Human T-cell leukemia virus 1 infection
Cellular Senescence
Oncogene Induced Senescence
Toll Like Receptor 10 (TLR10) Cascade
Toll Like Receptor 5 (TLR5) Cascade
MyD88 cascade initiated on plasma membrane
TRAF6 mediated induction of NFkB and MAP kinases upon TLR7/8 or 9 activation
MyD88 dependent cascade initiated on endosome
Toll Like Receptor 3 (TLR3) Cascade
Toll Like Receptor 7/8 (TLR7/8) Cascade
MyD88-independent TLR4 cascade
Toll Like Receptor 9 (TLR9) Cascade
TRIF (TICAM1)-mediated TLR4 signaling
MyD88:MAL(TIRAP) cascade initiated on plasma membrane
Toll Like Receptor TLR6:TLR2 Cascade
Toll Like Receptor TLR1:TLR2 Cascade
Toll Like Receptor 2 (TLR2) Cascade
Pathway
0 0.5 1 1.5 2 2.5
-log10 adjusted value
(B2)
ErbB signaling pathway
regulation of protein localization to plasma membrane
regulation of protein localization to cell periphery
positive regulation of striated muscle cell apoptotic process
positive regulation of cardiac muscle cell apoptotic process
Neurotrophin signaling pathway
regulation of protein localization to membrane
Wnt signaling pathway
Axon guidance
Lipid and atherosclerosis
Human T-cell leukemia virus 1 infection
Oncogene Induced Senescence
Cellular Senescence
TRIF (TICAM1)-mediated TLR4 signaling
TRAF6 mediated induction of NFkB and MAP kinases upon TLR7/8 or 9 activation
Toll Like Receptor TLR6:TLR2 Cascade
Toll Like Receptor TLR1:TLR2 Cascade
Toll Like Receptor 9 (TLR9) Cascade
Toll Like Receptor 7/8 (TLR7/8) Cascade
Toll Like Receptor 5 (TLR5) Cascade
Toll Like Receptor 3 (TLR3) Cascade
Toll Like Receptor 2 (TLR2) Cascade
Toll Like Receptor 10 (TLR10) Cascade
MyD88:MAL(TIRAP) cascade initiated on plasma membrane
MyD88-independent TLR4 cascade
MyD88 dependent cascade initiated on endosome
MyD88 cascade initiated on plasma membrane
1.6 1.8 2 2.2 2.4
-log10 adjusted value
Source
GO
KEGG
Reactome
Count
2
2.25
2.5
2.75
3
(A3)
Source GO KEGG Reactome
regulation of miRNA transcription
miRNA transcription
regulation of miRNA metabolic process
miRNA metabolic process
response to estradiol
Non-small cell lung cancer
PD-L1 expression and PD-1checkpoint pathway in cancer
Proteoglycans in cancer
Chemical carcinogenesis-receptor activation
Prolactin signaling pathway
Pancreatic cancer
EGFR tyrosine kinase inhibitor resistance
Diseases of signal transduction by growth factor receptors and second messengers
TFAP2 (AP-2) family regulates transcription of growth factors and their receptors
Signaling by ALK
Transcriptional regulation by the AP-2 (TFAP2) family of transcription factors
Signaling by PTK6
Signaling by Non-Receptor Tyrosine Kinases
Signaling by ERBB4
Pathway
0 1 2 3 4
-log10 adjusted value
(B3)
regulation of miRNA transcription
miRNA transcription
regulation of miRNA metabolic process
PD-L1 expression and PD-1 checkpoint pathway in cancer
Non-small cell lung cancer
miRNA metabolic process
response to estradiol
Diseases of signal transduction by growth factor receptors and second messengers
TFAP2 (AP-2) family regulates transcription of growth factors and their receptors
Proteoglycans in cancer
Chemical carcinogenesis-receptor activation
Signaling by ALK
Transcriptional regulation by the AP-2 (TFAP2) family of transcription factors
Signaling by PTK6
Signaling by Non-Receptor Tyrosine Kinases
Signaling by ERBB4
Prolactin signaling pathway
Pancreatic cancer
EGFR tyrosine kinase inhibitor resistance
2.5 3 3.5 4
-log10 adjusted value
Source
GO
KEGG
Reactome
Count
2
2.5
3
3.5
4

(A4)

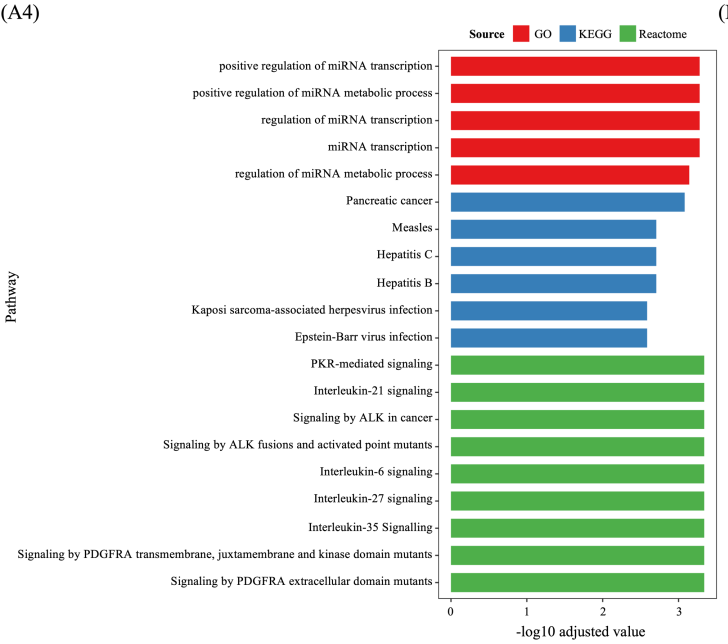

(B4)

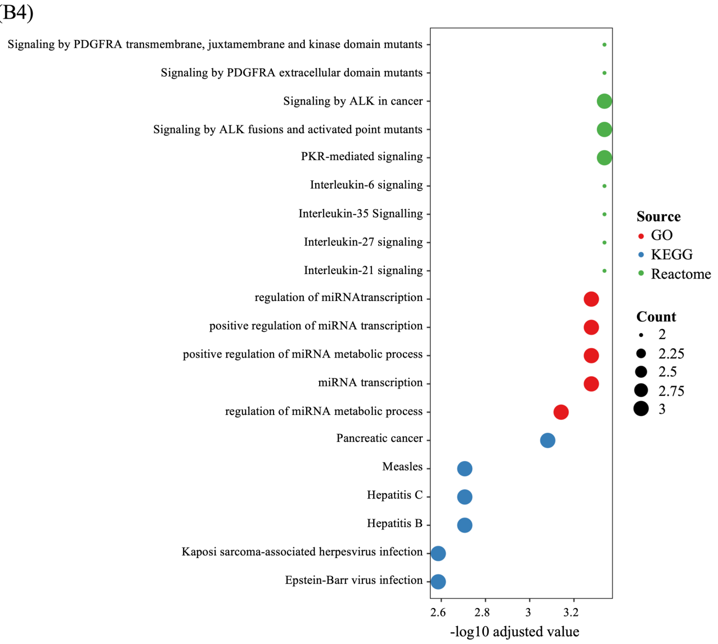

(A5)

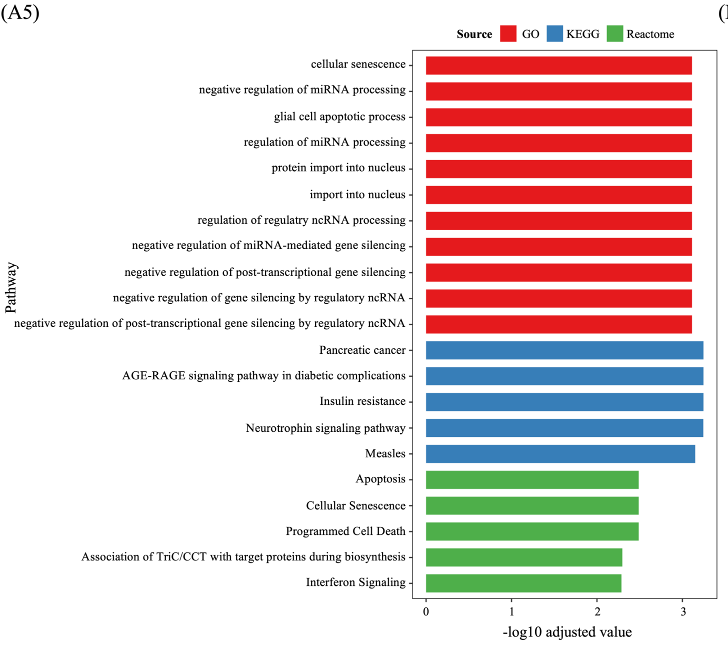

(B5)

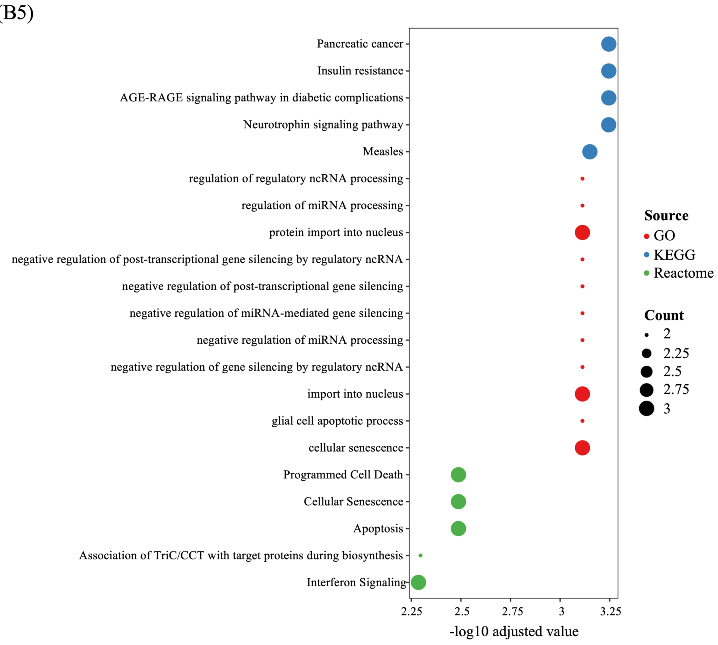

(A6)

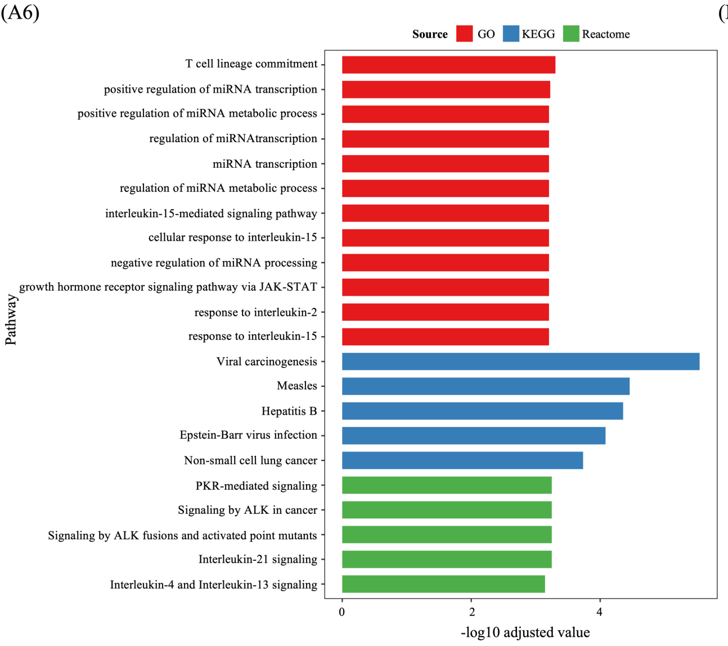

(B6)

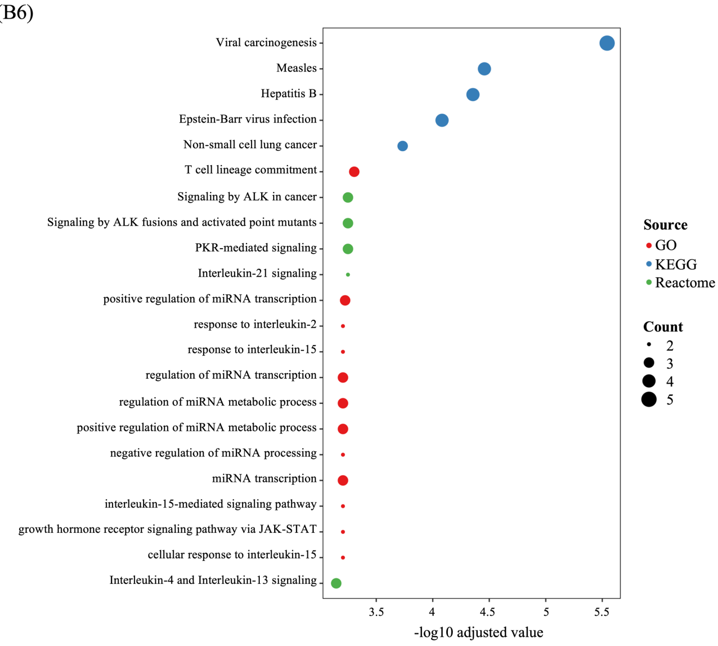

(A7)

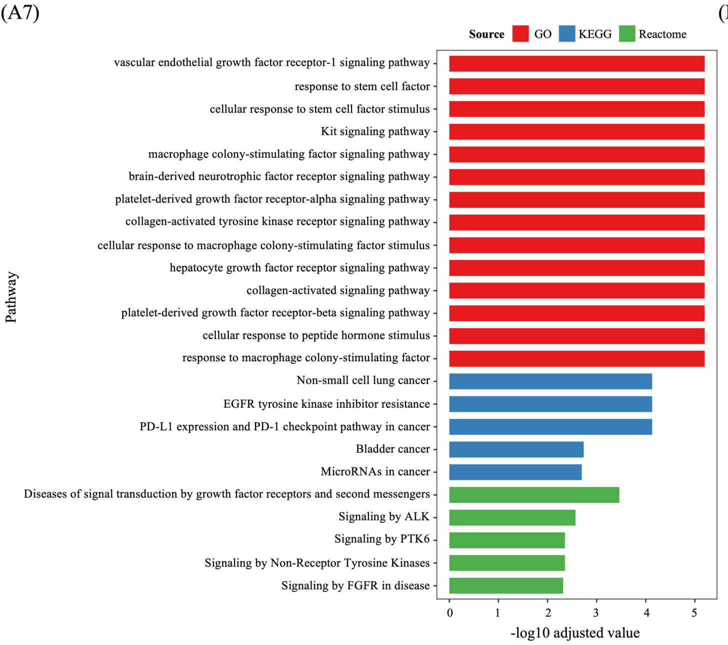

(B7)

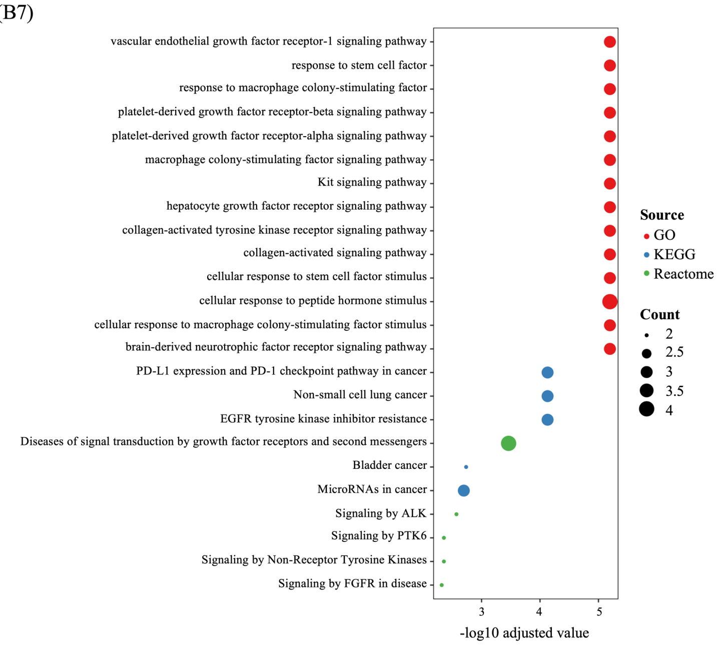

Supplementary figure 2: Pathway enrichment results for PD_LRRK2, PD_GBA, sporadic_PD, prodromal_GBA, prodromal_hyposmia, prodromal_RBD and SWEDD subgroup, respectively, with three colors representing three databases: GO, KEGG, and Reactome. The top five most significant pathways are displayed via (A) bar plot and (B) Unified dotplot, where dot size indicates gene counts, and the significance threshold, adjusted p-value, for each database was set at 0.05.
